# Significance of nutritional status in the development of periprosthetic infections: A retrospective analysis of 194 patients

**DOI:** 10.1007/s00132-020-03922-8

**Published:** 2020-05-18

**Authors:** Dirk Zajonz, Alexandros Daikos, Florian Prager, Melanie Edel, Robert Möbius, Johannes K. M. Fakler, Andreas Roth, Mohamed Ghanem

**Affiliations:** 1 Department of Orthopaedics, Traumatology and Plastic Surgery, University Hospital Leipzig, Liebigstraße 20, 04103 Leipzig, Germany; 2 ZESBO, Center for Research on Musculoskeletal Systems, University of Leipzig, Semmelweisstraße 14, 04103 Leipzig, Germany; 3 Clinic for Orthopaedics, Traumatology and Reconstructive Surgery, Zeisigwald Hospitals BETHANIEN Chemnitz, Zeisigwaldstraße 101, 09130 Chemnitz, Germany

**Keywords:** Vitamin deficiency, Protein deficiency, Malnutrition, Body mass index, Arthroplasty, Vitaminmangel, Eiweißmangel, Mangelernährung, Body-Mass-Index, Arthroplastik

## Abstract

**Background:**

Malnutrition caused by protein and vitamin deficiencies is a significant negative prognostic factor in surgical wound healing disorders and infections. Particularly in elective surgery, preoperative compensation of deficiencies is advisable to avoid negative postoperative consequences. This study examined the nutritional and protein balance of patients with periprosthetic hip and knee joint infections.

**Material and methods:**

Patients with periprosthetic hip or knee joint infections constituted the study group (SG). Control group I (CG I) included patients with primary implants and CG II included patients who required revision surgery because of aseptic loosening. Relevant nutritional and protein parameters were determined via analysis of peripheral venous blood samples. In addition, a questionnaire was used to evaluate the nutritional and eating patterns of all patients. The nutritional risk screening (NRS) 2002 score and body mass index (BMI) were also calculated for all participants.

**Results:**

Differences were found in the albumin level (SG: 36.23 ± 7.34, CG I: 44.37 ± 3.32, *p* < 0.001, CG II: 44.06 ± 4.24, *p* < 0.001) and total protein in serum (SG: 65.42 ± 8.66, CG I: 70.80 ± 5.33, *p* = 0.004, CG II: 71.22 ± 5.21, *p* = 0.004). The number of patients with lowered albumin levels (SG 19/61, CG I 1/78, CG II 2/55) and total protein in serum (SG: 12/61, CG I 5/78, CG II 2/55) also showed considerable variation. The number of patients with a NRS 2002 score ≥3 differed significantly between SG and both CGs (SG: 5/61, CG I 1/78, CG II 0/55); however, these differences could not be confirmed using BMI.

**Conclusion:**

As expected, lowered albumin and total protein levels were observed in PJI due to the acute phase reaction. The NRS can be performed to exclude nutritional deficiency, which cannot be excluded based on BMI. In cases of periprosthetic joint infection it is reasonable to compensate the nutritional deficiency with dietary supplements.

## Abstract (de)

**Hintergrund:**

Unterernährung aufgrund von Protein- und Vitaminmangel ist ein bedeutender negativer Prognosefaktor bei chirurgischen Wundheilungsstörungen und Infektionen. Insbesondere bei elektiven Eingriffen ist ein präoperativer Ausgleich von Mangelerscheinungen ratsam, um negative postoperative Folgen zu vermeiden. Ziel dieser Studie war es, die Ernährungs- und Proteinbilanz von Patienten mit periprothetischen Hüft- und Kniegelenkinfektionen zu untersuchen.

**Material und Methoden:**

Patienten mit periprothetischen Hüft- oder Kniegelenksinfektionen bildeten die Studiengruppe (SG). Die Kontrollgruppe I (CG I) umfasste Patienten mit Primärimplantaten, während die CGII Patienten einschloss, die wegen aseptischer Lockerung eine Revisionsoperation benötigten. Bei allen Patienten wurden relevante Ernährungs- und Proteinparameter durch die Analyse von Blutproben bestimmt. Darüber hinaus wurde ein Fragebogen zur Bewertung der Ernährungs- und Essgewohnheiten aller Patienten erhoben. Der Score des Nutritional Risk Screening (NRS) 2002 und der Body-Mass-Index (BMI) wurden ebenfalls für alle Teilnehmer bestimmt.

**Ergebnisse:**

Unterschiede wurden im Albuminspiegel (SG: 36,23 ± 7,34, CG I: 44,37 ± 3,32; *p* < 0,001; CG II: 44,06 ± 4,24, *p* < 0,001) und im Gesamtprotein im Serum festgestellt (SG: 65,42 ± 8,66, CG I: 70,80 ± 5,33; *p* = 0,004; CG II: 71,22 ± 5,21; *p* = 0,004). Die Anzahl der Patienten mit erniedrigten Albuminwerten (SG: 19/61, CG I: 1/78, CG II: 2/55) und des Gesamtproteins im Serum (SG: 12/61, CG I: 5/78, CG II: 2/55) zeigten ebenfalls beträchtliche Unterschiede. Die Anzahl der Patienten mit kritischem Ernährungsstatus (NRS-2002-Score ≥3) unterschied sich signifikant zwischen SG und beiden CG (SG: 5/61, CG I: 1/78, CG II: 0/55). Diese Unterschiede konnten jedoch anhand des BMI nicht bestätigt werden.

**Schlussfolgerung:**

Wie erwartet, werden aufgrund der Akute-Phase-Reaktion bei periprothetischen Infektionen (PPI) niedrigere Albumin- und Gesamtproteingehalte beobachtet. Mittels NRS kann ein Ernährungsmangel ausgeschlossen werden, dessen Beurteilung aufgrund des BMI nicht sicher möglich ist. Bei Auftreten einer periprothetischen Gelenkinfektion ist es sinnvoll, den Nährstoffmangel durch Nahrungsergänzungsmittel auszugleichen.

## Introduction

Despite the enormous progress in surgical treatment for reducing the risk of infections, periprosthetic joint infections (PJI) remain a major challenge and risk for the affected patients, the health system, and attending personnel [1–4]. Considering the grave effects of PJI on patients and the society, the optimization of patient-related risk factors is vital [4]. The effects of nutritional status and protein balance are considered to be significant prognostic factors for the development of PJI [5, 6]. Malnutrition is reported to be associated with complications, which range from prolonged hospital stay to impaired wound healing, after the hip and knee endoprosthesis implantation [5, 7–11]; however, this association seems to receive too little attention in current clinical practice. Jensen et al. reported that 50% of the patients who received elective total hip arthroplasty (THA) already had a clinical or subclinical nutrient deficiency before surgery [12]. The increasing supernutrition and the growing number of obese persons in industrial countries, which have caused an increase in metabolic diseases, support this finding. In contrast, Kaidar-Person et al. reported that obese persons can also be malnourished because their diets are often low in vitamins, proteins, and nutrients [13, 14]. In particular, low serum albumin level and protein deficiency are reportedly negative predictive factors with respect to the rates for perioperative and postoperative complications, such as PJI, associated with primary joint endoprostheses [15, 16]. Accordingly, a seven-fold increased risk of arthroplasty-related infections was observed at a preoperative albumin level of <35 g/L [9]; however, proteins are essential for adequate wound healing and serve as carriers for most vitamins (such as vitamin D and its metabolites), hormones, and mediators. The importance of vitamin D in orthopedic infections and particularly in septic disease progression has been proven [17–20]. It is also known that 99% of the vitamin D present in the human body exists in a protein-bound form (e.g. vitamin D-binding protein and albumin) and that it is fully effective only when the protein level is balanced [21, 22]. It is striking that none of the cited studies took the importance of the nutritional status, particularly the protein balance, into account.

This retrospective analysis aimed to examine the nutritional status of and the protein balance in patients with periprosthetic hip and knee joint infections. Patients with primary endoprosthesis implantation and aseptic loosening constituted the comparison groups.

## Material and methods

This study was approved by the ethics committee of the University of Leipzig (approval number: 025-16-01022016). The study was designed and the required sample size was estimated in advance using a power analysis program (G*Power; version 3.1.9.2., Axel Buchner, Edgar Erdfelder, Franz Faul, Albert-Georg Lang, Department of Psychology, Heinrich-Heine-University Düsseldorf, Germany). An effect size of medium value (d = 0.5) and a statistical test power of 80% (1 − β = 0.8) were assumed for detecting significant differences (α = 0.05).

This study was conducted at a university maximum care hospital in Europe and involved an analysis with patients of German nationality. The inclusion criteria for the study group (SG) were the presence of periprosthetic hip or knee joint infection, written consent for participation, and minimum age of 18 years. In the present study, periprosthetic infection was defined based on the criteria of the International Consensus Meeting on Periprosthetic Joint Infection (ICMPJI), 2013 [23, 24]. Patients with primary endoprosthesis implantation and revision surgery due to aseptic loosening served as the control groups I (CG I) and II (CG II), respectively. According to the matching procedure both the CGI and II were adapted to the SG with respect to age, body mass index (BMI), and implant location (THA or total knee arthroplasty, TKA). Aseptic loosening was defined as radiologically determined implant loosening with appropriate symptoms.

From 1 January 2015 to 29 September 2017, a total of 61 patients were included in the SG with 16/61 (26.2%) acute PJI and 45/61 (73.8%) low-grade PJI. Detailed information is given in Table 1. Acquisition of suitable patients for CGI and II (n_CG I_ = 78 and n_CG II_ = 55) was started at the same time but lasted until 3 November 2017. Total cholesterol, high-density lipoprotein (HDL) cholesterol, low-density lipoprotein (LDL) cholesterol, albumin, C‑reactive protein (CRP), hemoglobin, creatinine, and alanine aminotransferase levels as well as total protein in serum of all patients were determined via analysis of peripheral venous blood samples; all these levels were ascertained during inpatient admission prior to surgery. Furthermore, all patients completed a nutrition questionnaire comprising questions regarding eating habits, sports activities, and weight fluctuations. The nutritional risk screening (NRS) 2002 score and BMI were also calculated for all study participants [25].

**Table 1:** Comparison of the examination groups separated into acute and low-grade PJI with respect to laboratory tests

	Acute infection	Low-grade infection	*p*-value
Number of subjects (excluded antiosteoporotic treated patients)	26.2% (16/61)	73.8% (45/61)	–
Hemoglobin in mmol/l (7.2–10.0)	5.3 ± 1.53	6.5 ± 1.34	***0.015***
Leukocytes in 10^9^/l (3.5–9.8)	11.4 ± 4.3	7.6 ± 1.4	***<0.001***
C‑reactive protein in mg/l (< 5)	176.3 ± 64	27.3 ± 54	***<0.001***
Albumin in g/l (35–52)	28.0 ± 7.1	37.7 ± 5.5	***0.030***
Patients with lowered albumin values in %	80.0% (12/15)	36.4% (16/44)	***<0.001***
Serum protein in g/l (63–83)	59 ± 7.12	69.3 ± 6.32	***0.040***
Patients with lowered serum protein values in %	86.7% (13/15)	22.7% (10/44)	***<0.001***
Creatinine in µmol/l (45–84)	75 ± 36.1	77 ± 21.9	*0.390*
Alanine aminotransferase in µkat/l (0.17–0.58)	0.33 ± 0.13	0.32 ± 0.13	*0.700*

Pathological values are highlighted in italics

Statistically significant values are highlighted in bold

Values are expressed as median (± mean deviation) or as percentage (absolute/total)

Standard values or the physiological range are given in brackets

Data were statistically evaluated using Excel 2013 (Microsoft, Redmond, WA, USA) and SPSS v. 24.0 (IBM, Armonk, NY, USA). Data were reviewed for normal distribution using the Shapiro-Wilk test. The non-parametric Mann-Whitney U‑test was applied to compare metric scaled variables. Nominal and ordinal scaled variables were analyzed using the χ^2^-test or Fisherʼs test and *p* < 0.05 was considered to be statistically significant. Data were expressed as median and standard deviation (SD).

## Results

**Fig. 1:**
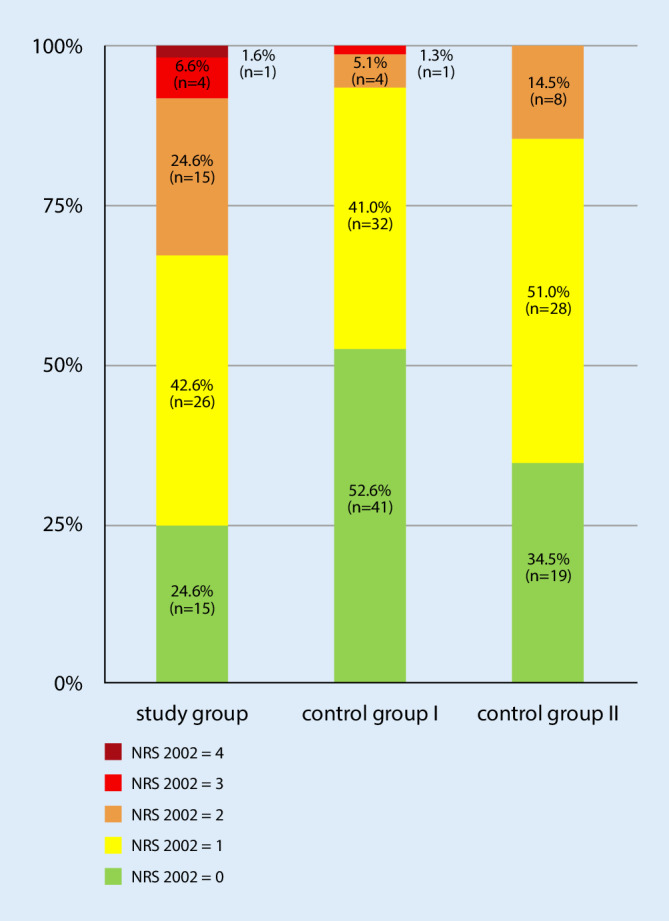
Nutritional risk screening (*NRS*) 2002 in the 3 groups: >3 nutritional risk points, preparation of a nutrition plan <3 points weekly repeated screening. If, for example, a large operation is planned for the patient a preventive nutrition plan should be pursued risk for PJI and wound healing disorders

**Fig. 2:**
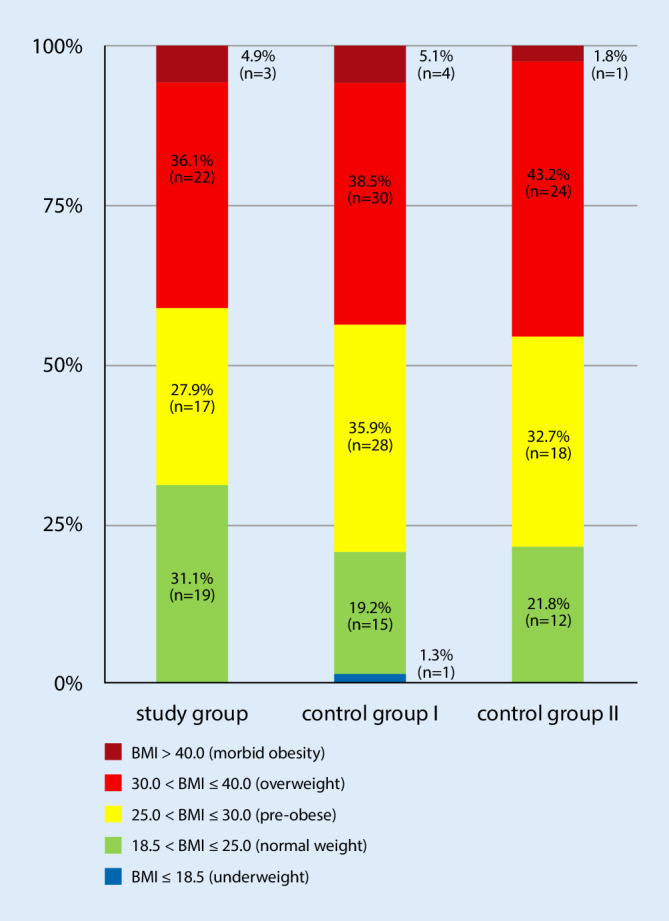
Body mass index (*BMI*) values in the 3 groups

Table 2 presents the characteristics of SG and CGs. A striking significant difference was observed in the NRS 2002 score between SG and CG I (SG: median = 1.0, SD = 0.9; CG I: median = 0.0, SD = 0.7, *p* < 0.001). A significant difference was also observed in the number of patients who exhibited a critical nutritional status (NRS 2002 score ≥3) between SG and both CGs (SG: 5/61, 8.2%; CG I: 1/78, 1.3%; CG II: 0/55, 0.0%). Fig. 1 shows the distribution of NRS 2002 scores within the individual groups; however, the differences found in NRS 2002 scores could not be confirmed using BMI, which showed no significant differences between the groups. Nevertheless, in all groups, the majority of the patients had an overweight BMI (Table 2; Fig. 2). The results of all laboratory tests as well as the laboratory-specific standard levels for each parameter are summarized in Table 3. The SG and both CGs showed significant differences in the hemoglobin (SG: 6.15 ± 2.05mmol/l, CG I: 8.56 ± 0.86mmol/l, *p* < 0.001, CG II: 8.18 ± 1.03mmol/l, *p* < 0.001) and CRP (SG: 86.21 ± 98.72mg/l, CG I: 4.55 ± 1.3 mg/l, *p* < 0.001, CG II: 13.69 ± 1.11mg/l, *p* < 0.001) levels. Significant differences were also found in total cholesterol levels, particularly HDL-cholesterol (SG: 1.04 ± 0.39mg/l, CG I: 1.62 ± 0.47mg/l, *p* < 0.001, CG II: 1.45 ± 0.46mg/l, *p* < 0.001) and LDL-cholesterol (SG: 2.65 ± 0.93mmol/l, CG I: 3.64 ± 1.18mmol/l, *p* < 0.001, CG II: 3.37 ± 0.94mmol/l, *p* < 0.001). The albumin levels (SG: 36.23 ± 7.34g/l, CG I: 44.37 ± 3.32g/l, *p* < 0.001, CG II: 44.06 ± 4.42g/l, *p* < 0.001) and total protein in serum (SG: 65.42 ± 8.66g/l, CG I: 70.80 ± 5.33g/l, *p* = 0.004, CG II: 71.22 ± 5.21g/l, *p* = 0.004) also significantly differed between CGs. A subanalysis of the available laboratory parameters of acute and low-grade PJI is shown in Table 1. In this context, it is noticeable that acute PJI showed significantly lower values concerning the albumin level and total protein in serum than low-grade PJI. With respect to the nutrition questionnaire responses, SG exhibited a significantly lower level of the daily time spent outdoors (SG: median = 1.0 h, SD = 1.15 h; CG I: median = 2.0 h, SD = 1.45 h, *p* = 0.006; CG II: median = 2.5 h, SD = 1.41 h, *p* < 0.001) or on the move (SG: median = 2.0 h, SD = 2.61 h; CG I: median = 3.0 h, SD = 5.35 h, *p* = 0.003 and CG II: median = 5.0 h, SD = 2.94 h, *p* < 0.001), i.e., for activities such as shopping or housework. Responses to the individual questions are presented in Table 4.

**Table 2:** Comparison of the examination groups (SG, CG I primary arthroplasty, CG II aseptic revision) with respect to the number of subjects, median age (min–max), number (absolute, %) of male subjects, THA or TKA BMI and NRS 2002

	SG	CG I	*p*-value (SG vs. CG I)	CG II	*p*-value (SG vs. CG II)
*General group characteristics*					
Number of subjects	61	78	–	55	–
Median age in years (min–max)	74 (35–88)	68 (46–87)	0.125	67 (21–84)	0.239
Number of male patients (%)	35 (57.4)	39 (50.0)	0.387	18 (32.7)	*0.008*
Number of THA (%)	32 (52.5)	48 (61.5)	0.282	28 (50.9)	0.868
Number of TKA (%)	29 (47.5)	30 (38.5)		27 (49.1)	
Median BMI in kg/m^2^ (SD)	27.8 (5.8)	28.6 (5.6)	0.337	28.7 (4.8)	0.652
Median nutritional risk screening (NRS 2002) score (SD)	1.0 (0.9)	0.0 (0.7)	*<0.001*	1.0 (0.7)	–
Patients with critical nutritional status (NRS ≥3) in %	8.2 (5/61)	1.3 (1/78)	n.a.	0.0 (0/55)	n.a.

*THA* total hip arthroplasty, *TKA* total knee arthroplasty, *BMI* body mass index, *SD* standard deviation, *SG* study group, *CGI* control group I, *CGII* control group II, *NRS* nutritional risk screening 2002 score, *n.a*. not available

**Table 3:** Comparison of the examination groups (SG, CG I primary arthroplasty, CG II aseptic revision) with respect to laboratory evaluations

	Laboratory-specific standard range	SG	CG I	*p*-value (SG vs. CG I)	CG II	*p*-value (SG vs. CG II)
*Laboratory test*						
Hemoglobin in mmol/l	7.2–10.0	6.16 ± 2.05	8.56 ± 0.86	*<0.001*	8.18 ± 1.03	*<0.001*
Leukocytes in 10^9^/l	3.5–9.8	8.30 ± 3.79	7.23 ± 1.57	0.243	7.41 ± 2.11	0.272
C‑reactive protein in mg/l	<5.0	86.21 ± 98.72	4.55 ± 1.39	*<0.001*	13.69 ± 1.11	*<0.001*
Cholesterol in mmol/l	<5.2	4.37 ± 1.08	5.78 ± 1.39	*<0.001*	5.25 ± 1.11	*<0.001*
High-density lipoprotein (HDL) cholesterol in mmol/l	>1.03	1.04 ± 0.39	1.62 ± 0.47	*<0.001*	1.45 ± 0.46	*<0.001*
Patients with lowered HDL cholesterol values in % (absolute)	<1.03	54.1 (33/61)	11.5 (9/78)	n.a.	23.6 (13/55)	n.a.
Low-density lipoprotein (LDL) cholesterol in mmol/l	<4.2	2.65 ± 0.93	3.64 ± 1.18	*<0.001*	3.37 ± 0.94	*<0.001*
Patients with increased LDL cholesterol values in % (absolute)	>4.2	3.3 (2/61)	28.2 (22/78)	n.a.	14.5 (8/55)	n.a.
Glucose in mmol/l	n.a.	7.77 ± 3.30	6.26 ± 2.12	*0.002*	6.12 ± 1.56	*0.001*
Noticeable glucose values in % (absolute)	<7.8	31.1 (19/61)	12.8 (10/78)	n.a.	12.7 (7/55)	n.a.
Albumin in g/l	35.0–52.0	36.23 ± 7.34	44.37 ± 3.32	*<0.001*	44.06 ± 4.24	*<0.001*
Patients with lowered albumin values in % (absolute)	<35.0	31.1 (19/61)	1.3 (1/78)	n.a.	3.6 (2/55)	n.a.
Total serum protein in g/l	63.0–83.0	65.42 ± 8.66	70.80 ± 5.33	*0.004*	71.22 ± 5.21	*0.004*
Patients with lowered serum protein values in % (absolute)	<63.0	19.7 (12/61)	6.4 (5/78)	n.a.	3.6 (2/55)	n.a.
Creatinine in µmol/l	45.0–84.0	83.80 ± 40.97	93.42 ± 85.43	0.599	75.98 ± 23.97	0.330
Alanine aminotransferase in µkat/l	0.17–0.58	0.34 ± 0.18	0.41 ± 0.23	0.074	0.37 ± 0.21	0.514

Statistically significant values are highlighted in italics

Values are expressed as median (± mean deviation) or as percentage (absolute/total)

Standard values or the physiological range are given in brackets

*SG* study group, *CGI* control group I, *CGII* control group II,

**Table 4:** Comparison of the examination groups (SG, CG I primary arthroplasty, CG II aseptic revision) with respect to questionnaire evaluation

	SG	CG I	*p*-value (SG vs. CG I)	CG II	*p*-value (SG vs. CG II)
*Questionnaire*					
Median time spending outdoors in hours per day (SD)	1.0 (1.15)	2.0 (1.45)	*0.006*	2.5 (1.41)	*<0.001*
Median time on the move (shopping, housework) in hours per day (SD)	2.0 (2.61)	3.0 (5.35)	*0.003*	5.0 (2.94)	*<0.001*
Sports activities in % (absolute)	19.7 (12/61)	30.8 (24/78)	0.138	38.2 (21/54)	*0.023*
Median sports activities in hours per week (SD)	3.5 (2.88)	2.5 (4.36)	0.149	3.0 (2.96)	0.484
Unintentional weight loss in the last 1–3 months in % (absolute)	11.5 (7/61)	3.8 (3/77)	0.088	12.7 (7/55)	0.836
Lower food intake in the last few weeks in % (absolute)	31.1 (19/61)	12.8 (10/78)	*0.008*	20.0 (11/54)	0.189

Statistically significant values are highlighted in italics

The values are expressed as median (± mean deviation) or as percentage (absolute/total)

The standard values or the physiological range are given in brackets

*SD* standard deviation, *SG* study group, *CGI* control group I, *CGII* control group II

## Discussion

The current context of social, economic, and political changes has led to a modification in the eating habits of individuals in industrialized countries and has raised discussions about the impact of these changes. Thus, overnutrition and increasing obesity in particular, pose a serious problem. The proportions of overweight (BMI ≥25.0 kg/m^2^) adult women increased from 22.2% (1974–1975) to 39.1% (1989) and 47.0% (1995–1996), corresponding to an increase of approximately 112% [25]. In this context, associated metabolic diseases, such as diabetes mellitus and heart diseases, and degenerative joint diseases, such as gonarthrosis and coxarthrosis, have gained in importance [26]. This study also demonstrated an increase in severity of obese nutritional status (BMI >30 kg/m^2^) (SG: 41.0%, CG I: 43.6%, and CG II: 45.5%) in all groups. If patients with a BMI of >25 kg/m^2^ are included, as much as 68.9%, 79.5%, and 78.2% of the patients in SG, CG I, and CG II, respectively, had a pre-obese or obese nutritional status (Fig. 2).

Clinically relevant vitamin or mineral deficiency is reported to be rare in western countries owing to the low cost and unlimited variety of available foods; however, many people consume enough food that is either unhealthy or of low nutritional value, i.e., food with a lack of proteins, vitamins, minerals, and fiber. In a series of publications, Kaidar-Person et al. reported that the prevalence of symptoms of deficiency of vitamins, proteins, etc. in the morbidly obese population was significantly higher than expected; [13, 14]; therefore, a high BMI with pre-obesity does not completely rule out the possibility of malnutrition. With respect to overweight patients, the survey results also revealed vitamin D and protein deficiencies in all groups (Table 3); in particular, patients in the SG exhibited significantly lower protein levels (i.e., albumin level and total protein in serum) than those in both CGs (Table 3). This finding can be attributed to the increased consumption during the acute phase reaction and is particularly evident in acute as well as in chronic PJI [27]. Whether a balancing of the proteins, which are consumed in the acute phase reaction is meaningful, cannot be proven based on this study. An isolated substitution of albumin in the course of infection, especially in sepsis, is controversially discussed [28]; however, studies have shown that reduced albumin levels are associated with an increased risk of orthopedic wound infections [29]. A further prospective study is planned for this purpose. Furthermore, optimal blood glucose control should be ensured to assess the effects of overnutrition and metabolic changes, such as prediabetes mellitus. In the present study, compared with those in the CGs a significantly higher number of patients in the SG showed abnormal blood glucose levels (Table 3). It has been proven that an increased level of glycated hemoglobin (HbA1c), in particular, is associated with an increased risk of PJI [30]. Determination and optimization of the level of HbA1c prior to elective endoprosthesis implantation is also advisable. Although a level of <7% is generally recommended, there is no international consensus on the ideal target level of HbA1c; however, in the short term this level can only be achieved to a limited extent in patients with severe diabetes [31, 32]. Therefore, optimal postoperative blood glucose control until adequate wound healing seems to be essential to avoid PJI [31].

A tendency with respect to conspicuous BMI could not be confirmed in the individual groups because there were no significant differences among them. In contrast, studies have reported that obesity is one of the most important modifiable patient factors in predicting PJI after THA or TKA; this is evidenced by data from a total of 10,690 and 9481 cases of primary THA and TKA, respectively, retrieved from the New Zealand Surgical Site Infection Improvement Program between 2013 and 2015 [33]. The negative influence of associated metabolic diseases, such as diabetes mellitus and circulatory disorders, on the development of PJI has been proven [34, 35]. Although a high BMI is reportedly associated with higher rates of wound healing disorders and PJI, an altered or decreased BMI is not a useful marker for malnutrition [4].

In the present study, NRS proved to be good predictive factor for PJI. Approximately 75.4% of the patients in the SG showed conspicuous NRS 2002 scores (NRS 2002 score ≥1). The exact distribution within the individual groups is shown in Fig. 1. Particularly in the field of spinal surgery and arthroplasty, the importance of NRS with respect to wound infections has been demonstrated [5, 27, 36]. A combination of NRS and preoperative analysis of the albumin levels and total protein in serum seems to be essential in this aspect [9, 27].

### Limitations

Within the scope of this study the consensus meeting criteria for defining PJI were used. This aspect could result in a lower detection of low-grade infections. Therefore, it would be sensible to use a more sensitive classification for future follow-up studies. A group-specific determination of HbA1c was not performed. In addition, the data are only a snapshot of the levels without a follow-up examination of the patients. For this reason, it is not possible to make statements about the previous nutritional and protein balance of the patients, especially before symptoms of PJI occurred. Therefore, it cannot be said whether they have contributed to the emergence of PJI. Finally, the outcome cannot be assessed according to the study parameters; however, a follow-up examination of the patients has been planned.

## Conclusion

**Fig. 3:**
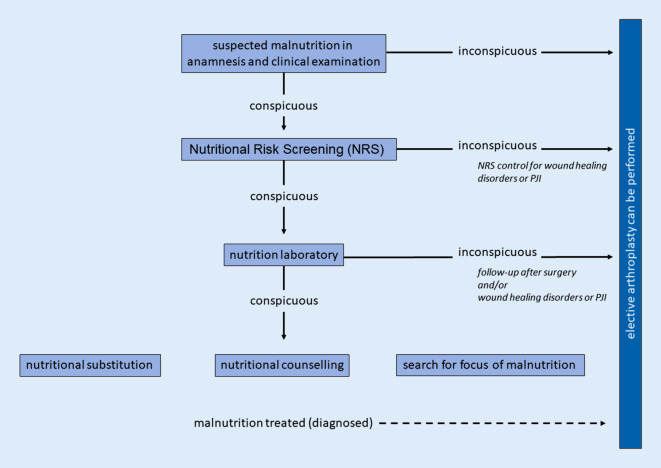
Diagnostic algorithm for assessing the nutritional status before elective arthroplasty

**Fig. 4:**
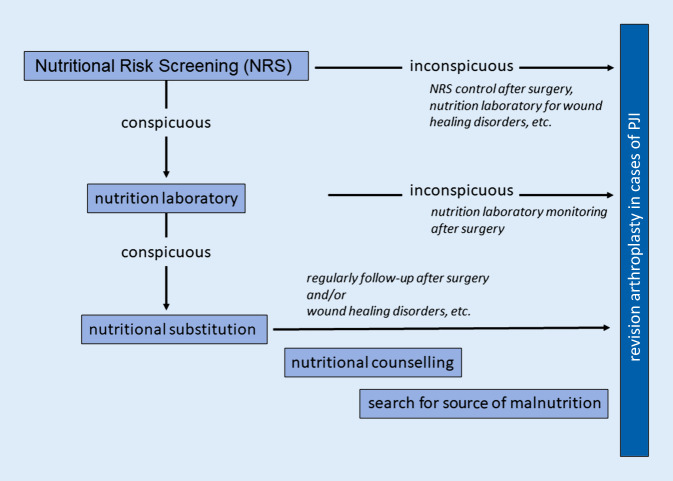
Diagnostic algorithm for assessing the nutritional status of periprosthetic joint infection (*PJI*)

To predict the risk for periprosthetic infections selected protein levels (e.g., albumin level and total protein in serum) should be preoperatively determined. In addition, NRS can be performed to exclude nutritional deficiencies. In contrast, BMI is not suitable for assessing nutritional deficiency because malnutrition can also be present in a pre-obese nutritional condition. In the case of an existing PJI, compensation for the nutritional deficiency with dietary supplements is recommended, particularly for protein and vitamin deficiencies. An algorithm for dealing with malnutrition in elective arthroplasty but especially in PJI should be followed. A procedure-related proposal for both scenarios is presented in Figs. 3 and 4, respectively.

## Abbreviations

BMI: Body mass index
CG I: Control group I
CG II: Control group II
CRP: C‑reactive protein
HbA1c: Glycated hemoglobin
HDL: High-density lipoprotein
ICMPJI: International consensus meeting on periprosthetic joint infection
LDL: Low-density lipoprotein
NRS: Nutritional risk screening
PJI: Periprosthetic joint infection
SD: Standard deviation
SG: Study group
THA: Total hip arthroplasty
TKA: Total knee arthroplasty

## References

[1] Adeli B, Parvizi J (2012). Strategies for the prevention of periprosthetic joint infection. J Bone Joint Surg Br.

[2] Boettner F, Cross MB, Nam D (2011). Functional and emotional results differ after aseptic vs septic revision hip Arthroplasty. HSS J.

[3] Hanssen AD, Osmon DR, Nelson CL (1997). Prevention of deep periprosthetic joint infection. Instr Course Lect.

[4] Zajonz D, Brand A, Lycke C (2018). Risk factors for early infection following hemiarthroplasty in elderly patients with a femoral neck fracture. Eur J Trauma Emerg Surg.

[5] Cross MB, Yi PH, Thomas CF (2014). Evaluation of malnutrition in orthopaedic surgery. J Am Acad Orthop Surg.

[6] Font-Vizcarra L, Lozano L, Ríos J (2011). Preoperative nutritional status and post-operative infection in total knee replacements: a prospective study of 213 patients. Int J Artif Organs.

[7] Del Savio GC, Zelicof SB, Wexler LM (1996). Preoperative nutritional status and outcome of elective total hip replacement. Clin Orthop Relat Res.

[8] Gherini S, Vaughn BK, Lombardi AV (1993). Delayed wound healing and nutritional deficiencies after total hip arthroplasty. Clin Orthop Relat Res.

[9] Greene KA, Wilde AH, Stulberg BN (1991). Preoperative nutritional status of total joint patients. Relationship to postoperative wound complication. J Arthroplasty.

[10] Huang R, Greenky M, Kerr GJ (2013). The effect of malnutrition on patients undergoing elective joint arthroplasty. J Arthroplasty.

[11] Ong KL, Kurtz SM, Lau E (2009). Prosthetic joint infection risk after total hip arthroplasty in the Medicare population. J Arthroplasty.

[12] Jensen JE, Smith TK, Jensen TG (1981). The Frank Stinchfield Award Paper. Nutritional assessment of orthopaedic patients undergoing total hip replacement surgery. Hip.

[13] Kaidar-Person O, Person B, Szomstein S (2008). Nutritional deficiencies in morbidly obese patients: a new form of malnutrition? Part A: vitamins. OBES SURG.

[14] Kaidar-Person O, Person B, Szomstein S (2008). Nutritional deficiencies in morbidly obese patients: A new form of malnutrition? Part B: minerals. OBES SURG.

[15] Bohl DD, Shen MR, Kayupov E (2016). Is Hypoalbuminemia associated with septic failure and acute infection after revision total joint Arthroplasty? A study of 4517 patients from the national surgical quality improvement program. J Arthroplasty.

[16] Kamath AF, Nelson CL, Elkassabany N (2017). Low albumin is a risk factor for complications after revision total knee Arthroplasty. J Knee Surg.

[17] Abhimanyu CAK (2017). The role of UV radiation and vitamin D in the seasonality and outcomes of infectious disease. Photochem Photobiol Sci.

[18] Hegde V, Dworsky EM, Stavrakis AI (2017). Single-dose, preoperative vitamin-D supplementation decreases infection in a mouse model of Periprosthetic joint infection. J Bone Joint Surg Am.

[19] Maier GS, Horas K, Seeger JB (2014). Is there an association between periprosthetic joint infection and low vitamin D levels?. Int Orthop.

[20] Signori V, Romanò CL, de Vecchi E (2015). May osteoarticular infections be influenced by vitamin D status? An observational study on selected patients. BMC Musculoskelet Disord.

[21] Cranney A, Horsley T, O’Donnell S (2007). Effectiveness and safety of vitamin D in relation to bone health. Evid. Rep. Technol. Assess..

[22] Feindt E, Ströder J (1977). Zur antimikrobiellen Wirkung von Vitamin D (Studies on the antimicrobial effect of vitamin D (author’s transl)). Klin Wochenschr.

[23] Fayaz HC, Jupiter JB (2017). The zeitgeist of challenging the evidence. A perspective on the international consensus meeting on Periprosthetic joint infection. Arch Bone Jt Surg.

[24] Honkanen M, Jämsen E, Karppelin M (2017). Concordance between the old and new diagnostic criteria for periprosthetic joint infection. Infection.

[25] Souza NPd, Lira PICd, Fontbonne A (2017). A (des)nutrição e o novo padrão epidemiológico em um contexto de desenvolvimento e desigualdades ((Mal)nutrition and the new epidemiological trend in a context of development and inequalities). Cien Saude Colet.

[26] Shin D, Kongpakpaisarn K, Bohra C (2018). Trends in the prevalence of metabolic syndrome and its components in the United States 2007–2014. Int J Cardiol.

[27] Yi PH, Frank RM, Vann E (2015). Is potential malnutrition associated with septic failure and acute infection after revision total joint arthroplasty?. Clin Orthop Relat Res.

[28] Vincent J-L, Russell JA, Jacob M (2014). Albumin administration in the acutely ill: what is new and where next?. Crit Care.

[29] Yuwen P, Chen W, Lv H (2017). Albumin and surgical site infection risk in orthopaedics: a meta-analysis. BMC Surg.

[30] Shohat N, Muhsen K, Gilat R (2018). Inadequate Glycemic control is associated with increased surgical site infection in total joint Arthroplasty: a systematic review and meta-analysis. J Arthroplasty.

[31] Adams AL, Paxton EW, Wang JQ (2013). Surgical outcomes of total knee replacement according to diabetes status and glycemic control, 2001 to 2009. J Bone Joint Surg Am.

[32] Giori NJ, Ellerbe LS, Bowe T (2014). Many diabetic total joint arthroplasty candidates are unable to achieve a preoperative hemoglobin A1c goal of 7 % or less. J Bone Joint Surg Am.

[33] Jung P, Morris AJ, Zhu M (2017). BMI is a key risk factor for early periprosthetic joint infection following total hip and knee arthroplasty. N Z Med J.

[34] Cunningham DJ, Kavolus JJ, Bolognesi MP (2017). Common medical Comorbidities correlated with poor outcomes in hip Periprosthetic infection. J Arthroplasty.

[35] Shohat N, Goswami K, Tarabichi M (2018). All patients should be screened for diabetes before total joint Arthroplasty. J Arthroplasty.

[36] Rai J, Gill SS, Kumar BRJS (2002). The influence of preoperative nutritional status in wound healing after replacement arthroplasty. Orthopedics.

